# Decomposition process of copper(ii) formate tetrahydrate through molecular dynamics simulations

**DOI:** 10.1039/d6ra07869a

**Published:** 2026-10-16

**Authors:** Nihesh Mohan, Juan Ignacio Ahuir-Torres, Hiren Kotadia, Fosca Conti, Gordon Elger

**Affiliations:** a Institute of Innovative Mobility (IIMo), Technische Hochschule Ingolstadt Esplanade 10 Ingolstadt 85049 Germany nihesh.mohan@thi.de; b School of Engineering and Built Environment, Liverpool John Moores University Liverpool L3 3AF UK; c Department of Chemical Sciences, University of Padova Padova 35131 Italy fosca.conti@unipd.it

## Abstract

The thermal decomposition of Cu(ii) formate tetrahydrate into metallic Cu is a multi-stage process critical to the development of particle-free conductive inks. This work correlates macroscopic thermal behavior with atomic-scale transformations by integrating physicochemical analyses with reactive molecular dynamics simulations. Differential scanning calorimetry reveals three distinct dehydration peaks at 52 °C, 80 °C and 102 °C followed by a final decomposition at 217 °C that reduces Cu formate into metallic Cu. Simulations use reactive force field (ReaxFF) to track step-wise dehydration and associated charge distribution dynamics. The simulations demonstrate that the loss of the first three hydration water molecules occurs from the outer lattice, leaving the Cu^2+^ coordination sphere intact. However, at 217 °C, the release of the final, strongly bound water molecule destabilizes the formate–copper interactions, acting as a stoichiometric structural trigger for the reduction process to Cu^0^. These findings offer frameworks to model decomposition kinetics in Cu complexes and optimize the corresponding inks for applications in flexible printed electronics.

## Abstract 2

The thermal decomposition of Cu(ii) formate tetrahydrate into metallic Cu is a multi-stage process critical to the development of particle-free conductive inks.

## Graphical abstract

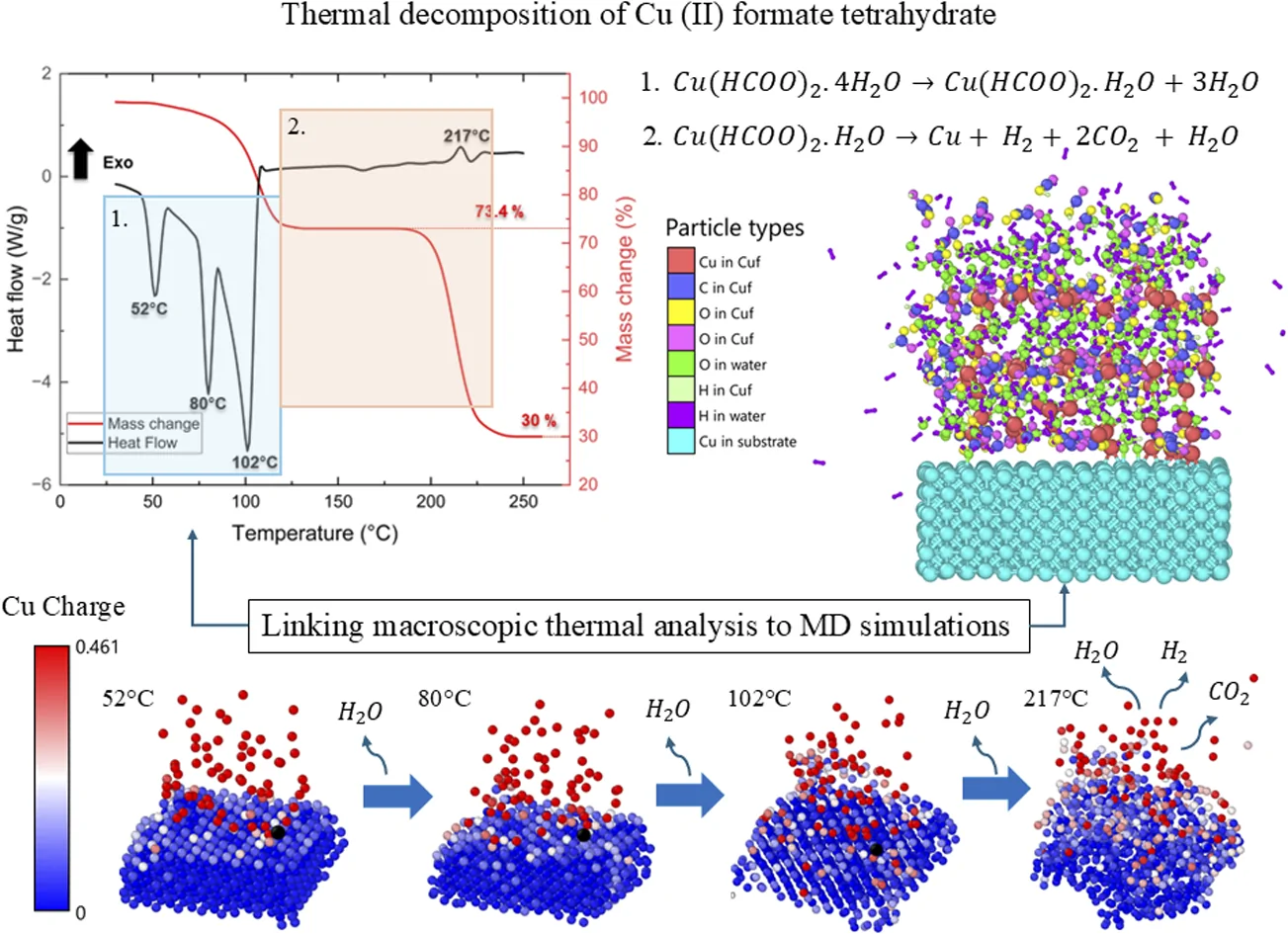

## Introduction

Flexible printed electronics (FPE) require high-performance conductive inks compatible with flexible substrates. Among these conductive inks, Cu-based ones are very promising. While Cu offers electrical and thermal conductivities comparable to Ag and Au at a lower cost, its application is hindered by its susceptibility to oxidation under ambient conditions and the agglomeration of nanoparticles (NPs) during storage, leading to limited shelf life.^1–3^ To mitigate this issue, the Cu particles are typically coated with surface capping agents (such as polyvinylpyrrolidone or oleylamine). However, removing this coating requires elevated processing temperatures (>200 °C) to enable diffusion-assisted sintering.^3–5^ Substrates for FPE are often characterized by low degradation temperature; for instance, polyethylene terephthalate (PET) degrades at *ca.* 175 °C.^6^ These problems restrict the substrate choice and its scalability for application in FPE. To address these limitations, metal–organic decomposition (MOD) or precursor-derived particle-free inks can be used. A typical example of the ionic Cu precursors that form stable Cu^2+^ complexes is Cu(ii) formate tetrahydrate (Cuf) that can be dissolved in complexing solvents (including alkanolamines, alkylamines, pyridines).^7–14^ These complexes enable the *in situ* formation of Cu NPs during low-temperature thermal decomposition (<150 °C), thereby releasing by-products (such as H_2_, H_2_O, CO_2_).^11,12^ Cuf follows a two-step decomposition process, as shown in eqn (1) and (2).^14–17^

1 Cu(HCOO)_2_·4H_2_O → Cu(HCOO)_2_·H_2_O + 3H_2_O

2 Cu(HCOO)_2_·H_2_O → Cu + H_2_ + 2CO_2_ + H_2_O

Despite these advantages, the design of Cu MOD inks remains empirical, as the molecular-scale mechanisms governing dehydration, ligand reorganisation and Cu^2+^ to Cu^0^ reduction remain unclear. Previous kinetic and thermodynamic studies by Galwey *et al.* and Mohamed *et al.* have thoroughly characterised the thermal decomposition of anhydrous Cu(ii) formate under various atmospheres.^18,19^ However, these studies focus only on the anhydrous state and the transient coordination changes, while the multi-stage dehydration mechanism of Cuf remain unclear at atomistic level. Recent research has focused on formulating Cuf with various amine ligands to achieve self-reducing MOD inks.^20^ Choi *et al.* and Marchal *et al.* have demonstrated that ligand concentration and denticity play important roles in phase evolution (transition from molecular precursor Cuf to metallic Cu), and also in film densification, and oxidation protection.^21,22^ Nabi *et al.* further discussed macroscopic aspects of thermal decomposition processes, emphasizing on how precursor chemistry dictates low-temperature metallization.^23^ To mechanistically understand these processes, researchers have used *in situ* X-ray spectroscopy to identify transient Cu(i) intermediates during thermal decomposition.^24^ Shin *et al.* also used density functional theory (DFT) to map the thermochemistry of bis(amino)Cu formate formulations.^25^ While these studies elucidate the role of organic ligands and intermediate oxidation states, they primarily rely on macroscopic spectroscopy or static, gas-phase DFT models. The foundational, dynamic atomic-level transition of the base precursor (Cuf) specifies, how the disassembly of the Cuf crystal lattice and stepwise loss of water of hydration actively trigger the initial structural and electronic change remains unexplored. In previous study, macroscopic techniques such as differential scanning calorimetry (DSC) and thermogravimetric analysis (TGA) of Cuf have identified distinct endothermic dehydration stages at 52 °C, 80 °C and 102 °C, followed by decomposition at ∼217 °C, with ∼30% mass loss from hydration water and 73% total mass loss to metallic Cu.^14,15^

This study uses Molecular Dynamics (MD) simulations to investigate the decomposition of Cuf, bridging the gap between macroscopic thermal behaviour and atomic-scale transformations. A 3D crystal lattice of Cuf is realised using the 2D lattices present in the literature.^26,27^ MD simulations are then performed explicitly at the critical transition temperature, involving dehydration and decomposition identified by thermal analyses.^28,29^ Crucially, these simulations utilize a reactive force field (ReaxFF), which enables the dynamic modelling of the continuous formation and destruction of all chemical bond types during the reaction. The atomistic approach enables real-time tracking of step-wise dehydration, Cu coordination transitions, and dynamic charge equilibrium to quantify the Cu^2+^ to Cu^0^ reduction. These simulations also reveal how the release of the tightly bound fourth water molecule destabilizes the formate ligands and acts as a stoichiometric structural trigger for the final decomposition into metallic Cu. The insights from the Cuf decomposition at atomic scale establish a framework for developing further models and understanding the decomposition mechanisms in Cuf-based MOD inks.

## Results and discussion

### Thermal boundary conditions and experimental validation

The decomposition process of Cuf initiates with the evaporation of three of the four water of crystallization molecules structurally bound to the Cu–formate complex per formula unit, as shown in the DSC and TGA profiles (Fig. 1a). This is observed in the three distinct endothermic peaks at 52 °C, 80 °C, and 102 °C, respectively. After the evaporation of the three water molecules, a mass loss of 26.6 wt% is observed from the TGA analysis which closely fits the calculated theoretical mass loss from eqn (1). This is further confirmed by the mass spectrum (MS) *via* Quasi-Multiple Ion Detection (QMID) (Fig. 1b), which shows a broad, dominant evolution of water molecules peaking at 107 °C during the exact temperature window as that of DSC/TGA curves, *i.e.*, with no concurrent release of CO_2_ or H_2_ at this stage. Following this intermediate partially dehydrated stage, a small, exothermic decomposition peak is observed at 217 °C in the DSC curve (Fig. 1a). It corresponds to a secondary mass-loss step, yielding a final residual mass of ∼30 wt% as observed in the TGA curve (Fig. 1a). While residual mass alone is not definitive proof of elemental purity, this secondary mass-loss step closely aligns with the theoretical atomic weight ratio of pure Cu to the initial Cuf precursor. Crucially, this is supported by the concurrent MS profiles (Fig. 1b), which confirm the complete evolution of all expected organic byproducts (CO_2_, H_2_O and H_2_) at this stage without further residual outgassing, validating the basis for the complete reduction modeled in the subsequent simulations. The MS profiles perfectly captured the complex decomposition process, as shown in eqn (2), where a sharp release of CO_2_ is observed at 221 °C, accompanied by the concurrent evolution of the final, strongly bound fourth H_2_O crystallization molecule (221 °C) and H_2_ gas (223 °C).

**Fig. 1:**
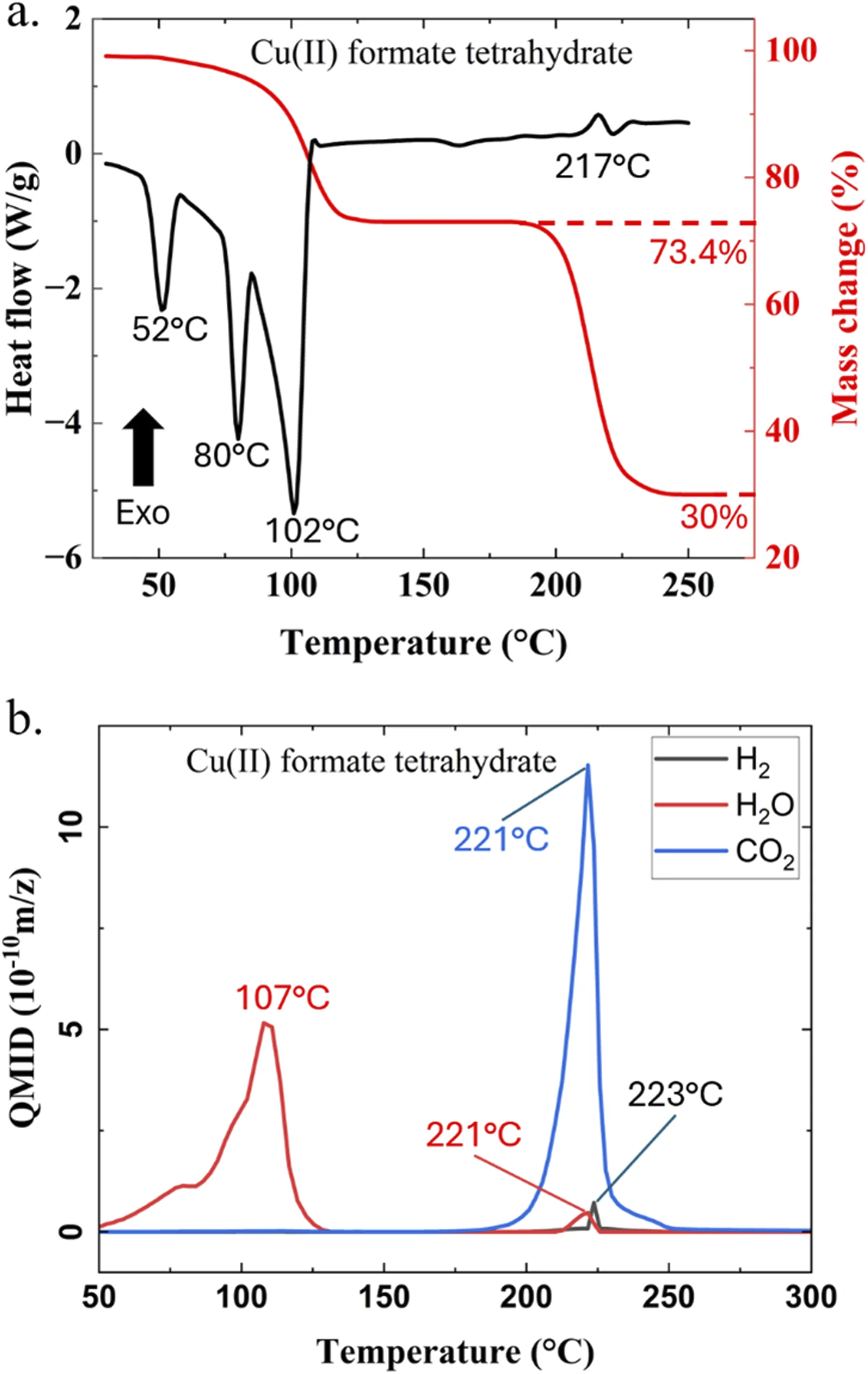
Thermal analyses (DSC/TGA) and mass spectrum (MS) of Cuf. (a) Differential Scanning Calorimetry (DSC, black curve) and Thermogravimetric Analysis (TGA, red curve) profiles. The data reveal a three-stage endothermic dehydration process below 120 °C (resulting in a 73.4% residual mass), followed by a final decomposition at 217 °C, yielding a final mass of ∼30%, corresponding to pure metallic Cu. (b) Mass spectrum tracking the relative evolution of H_2_O (red), CO_2_ (blue), and H_2_ (black) gases *via* Quasi-Multiple Ion Detection (QMID), confirming water loss during the initial heating stages and the massive release of CO_2_ and H_2_ during the final decomposition step.

The results show that the final reduction to metallic Cu is linked to the release of the final hydration water. Consequently, the experimentally derived peak temperatures (52 °C, 80 °C, 102 °C, and 217 °C) were extracted and utilized as the explicit thermal boundary conditions for the dynamic MD simulations. To track the stepwise dehydration process and Cu charge distribution, MD simulations were performed at four distinct temperature steps corresponding to the endothermic and exothermic peaks observed in the DSC profile, as summarized in Table 1.

**Table 1:** Summary of the MD simulation stages mapped for the macroscopic thermal events of Cuf

MD simulation stage	*T* (K/°C)	Description of the process
Initialization	298 K (25 °C)	Conversion of 2D lattice to 3D monoclinic crystal; construction of the Cuf/Cu-substrate material model; baseline equilibration
Stage-1 (411–8400 ns)	325 K (52 °C)	Initial dehydration: observe and validate the desorption and release of the first crystallization water molecule from the Cuf complex
Stage-2 (8400–16 400 ns)	353 K (80 °C)	Second dehydration: observe and validate the release of the second crystallization water molecule
Stage-3 (16 400–24 400 ns)	375 K (102 °C)	Third dehydration: observe and validate the release of the third crystallization water molecule
Stage-4 (24 400–31 400 ns)	490 K (217 °C)	Final decomposition: observe the release of the tightly bound fourth crystallization water molecule and the subsequent decomposition of the Cuf complex into pure Cu

### Atomistic tracking of stepwise dehydration and decomposition in Cuf complex

To provide an atomistic explanation for the macroscopic endothermic peaks observed during thermal analysis, simulations were used to continuously track the physical desorption and spatial diffusion of H_2_O molecules from the Cuf crystal lattice (Fig. 2). The initial 3D Cuf supercell containing 63 Cu(HCOO)_2_ formula units and a corresponding stoichiometric pool of 252 hydration water molecules was constructed to represent Cuf on a metallic Cu. At 325 K (52 °C), localised thermal fluctuations provided sufficient kinetic energy to break the weakest intermolecular hydrogen bonds, primarily at the crystal–vacuum interface (see Fig. S1, showing MD image during the water desorption).

**Fig. 2:**
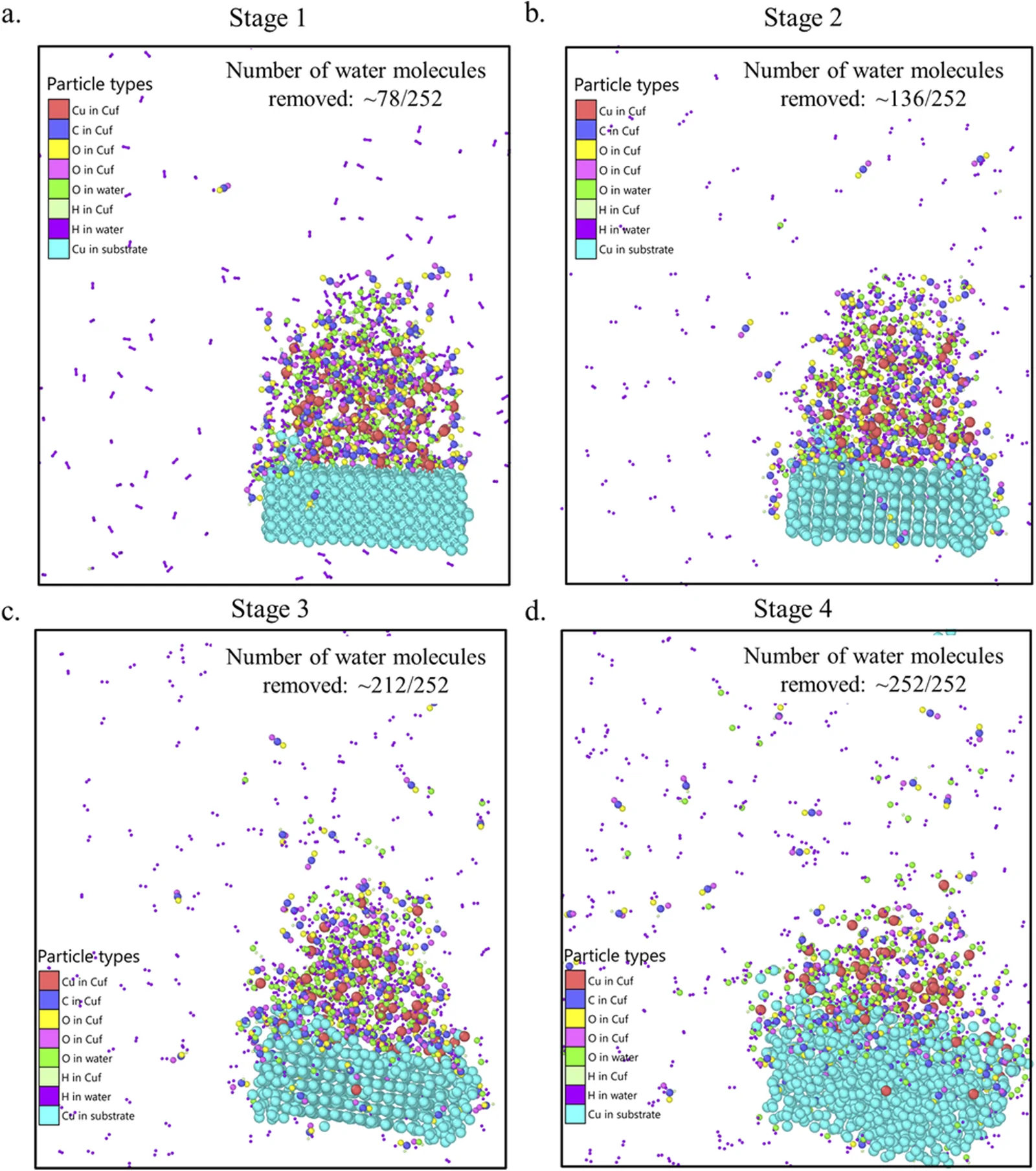
Atomistic snapshots from MD simulations tracking the stepwise dehydration and structural decomposition of Cuf on a Cu substrate. (a) At 325 K (52 °C), the initial hydration shell begins to desorb (∼78/252 H_2_O removed) while the main crystal lattice remains ordered. (b) At 353 K (80 °C), continued thermal excitation removes secondary hydration layers (∼136/252 H_2_O removed). (c) At 375 K (101 °C), the release of three-quarters of the hydration water (∼212/252 H_2_O removed) induces initial volumetric deformation of the intermediate monohydrate lattice. (d) At the decomposition onset of 490 K (217 °C), the forced release of the final, strongly bound inner-sphere water molecules (252/252 H_2_O removed) triggers a complete structural collapse of the crystal lattice, ligand dissociation and subsequent reduction.

Fig. 2a shows the release of approximately 78 water hydration molecules as a result of this thermal process. The result corresponds to a loss of roughly one stoichiometric equivalent of H_2_O per Cu centre (∼1/4 of the total), while the bulk monoclinic lattice of the Cuf crystal remained structurally coherent. At 353 K (80 °C), the thermal kinetic energy further increased, overcoming the secondary lattice binding forces and breaking the hydrogen-bonded network that propagated deeper into the bulk crystal. This increased the total number of H_2_O molecules removed to approximately 136, or roughly two stoichiometric equivalents of H_2_O per Cu centre (∼1/2 of the total) (Fig. 2b). At 375 K (102 °C), simulations recorded the expulsion of approximately 212 molecules, corresponding to the release of the third stoichiometric equivalent of H_2_O per Cu centre (∼3/4 of the total). This induces initial volumetric deformation as seen in Fig. 2c, causing the Cuf crystal to warp and contract into an intermediate monohydrate lattice (see Fig. S2, showing MD image comparing the tetrahydrate and monohydrate structure after dehydration). Despite this deformation and loss of nearly 80% of the crystallization water, the primary inner coordination sphere binding the central Cu^2+^ ions to the formate ligands remains largely intact. The results indicate that the release of first three water molecules is a chemical desorption process that causes initial geometric deformation of the intermediate monohydrate lattice but is not sufficient to electronically or sterically destabilise the core Cu^2+^ structure.

Upon reaching 490 K, sufficient thermal energy was achieved to break the Cu⋯O coordination interaction, causing the desorption of the strongly bound fourth H_2_O molecule. As a result, the central Cu^2+^ cation abruptly loses its stable coordination geometry. The neighbouring formate (HCOO^−^) anionic ligand now faces steric and electronic instability, causing it to dissociate from the Cu centre and undergo rapid chemical reaction. The previously ordered monohydrate Cuf crystal now completely dissociates, leading to a simultaneous release of CO_2_ and H_2_ gases (as seen in Fig. 2d). This also correlates with the observations from MS at the same temperature.

### Thermodynamic energy transitions during stepwise dehydration and decomposition

To elucidate the thermodynamic driving forces governing the structural transitions of the Cuf system, the system energy evolution was monitored across the entire microcanonical/canonical heating trajectory. The time evolution of the kinetic energy (Fig. 3b) which act as a direct proxy for system temperature demonstrates strict adherence to the prescribed stepwise thermal process from 298 K to 490 K (as indicated in Table 1).

**Fig. 3:**
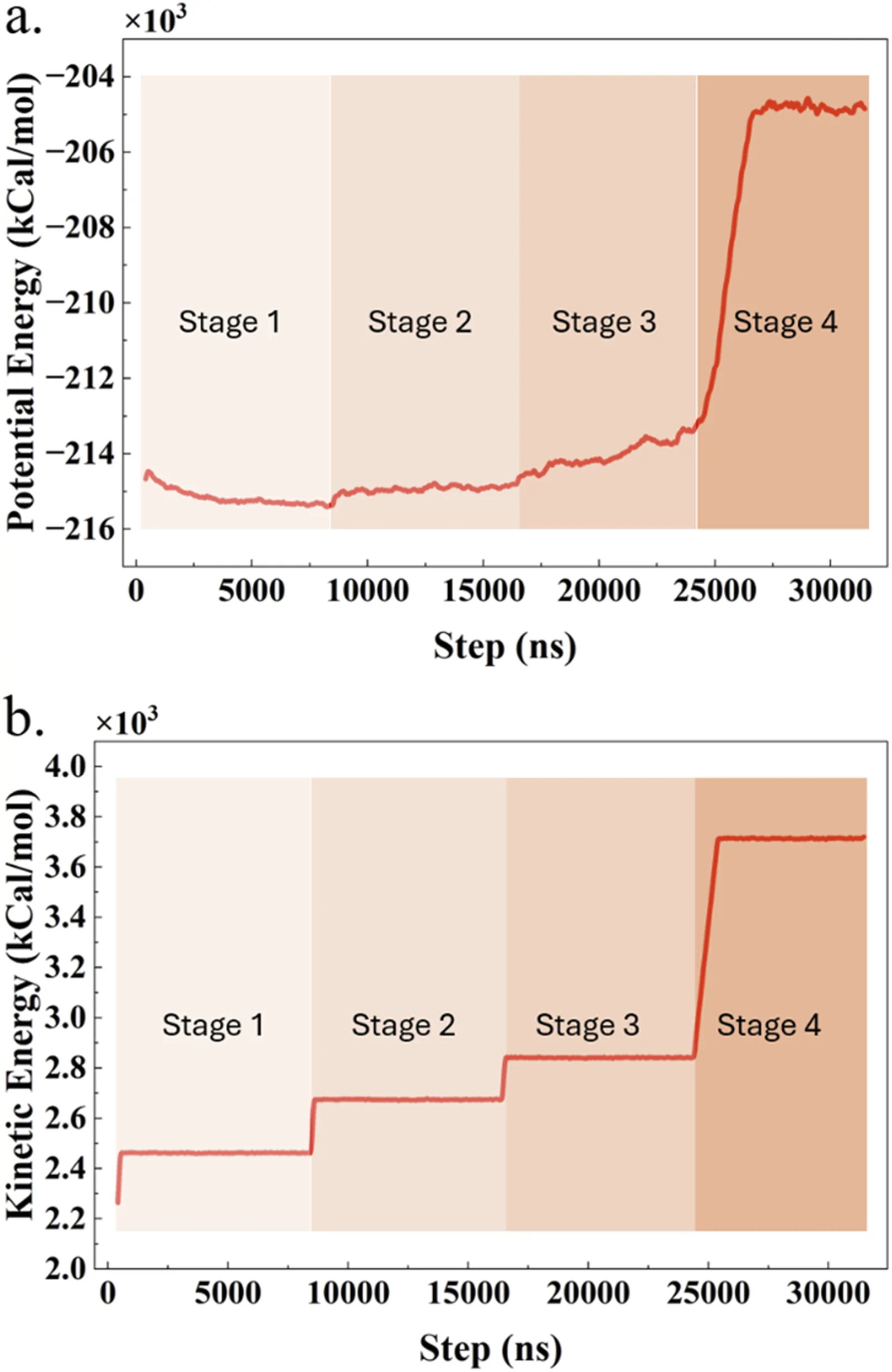
Thermodynamic profiles of the Cuf during the step-wise MD simulation across different stages. (a) Potential energy profile illustrating thermodynamic stability during the sequential dehydration processes, followed by a sharp asymptotic increase at 490 K (stage 4) that marks the decomposition of Cuf crystal. (b) Kinetic energy plot confirming the stable execution and equilibration of the system across the sequential heating protocol at 325 K, 353 K, 375 K, and 490 K.

The distinct plateaus in kinetic energy confirm that the system achieved localized thermodynamic equilibrium with each discrete heating window. During the primary desorption regimes spanning stage 1 to stage 3 (325 K to 375 K), the potential energy profile (Fig. 3a) exhibits a gradual increase. It should be noted that the negative values of the potential energy throughout these stages denote a bound thermodynamic state, indicating that these attractive intermolecular forces restrict the mobility of constituent molecules within the crystal lattice.^30^ In this zone, the influx of thermal energy is partitioned into intramolecular structural relaxation and isotropic dilatation of the monoclinic unit cell. The progressive increase in potential energy quantifies the energetic cost required to sequentially disrupt the peripheral, intermolecular hydrogen-bonding networks anchoring the outer-envelope aquo-ligands. However, the primary coordination sphere of the core Cu remains structurally unperturbed.

As observed in the thermodynamic profiles, the onset of the final fourth MD simulation stage at the critical temperature of 490 K corresponds to the step at 25 000 ns. Upon surpassing this critical thermal activation threshold at 490 K (217 °C), the system undergoes a strong thermodynamic divergence characterized by an abrupt, non-linear increase in the potential energy that subsequently flattens into a new stable plateau (Fig. 3a). This sharp bifurcation on the potential energy marks a clear transition from chemical water desorption to an irreversible thermochemical decomposition. While the subsequent plateau confirms the system has reached a new thermodynamic equilibrium corresponding to fully decomposed state. The energetic singularity captures the collective breakage of the high-energy inner-sphere coordination bonds, leading to a complete collapse of long-range crystalline order. The thermodynamic destabilization triggers the definitive multi-body molecular disintegration of the formate framework, providing clear atomistic evidence of the localized phase transition that drives the *in situ* transformation of Cuf to Cu^0^. To deconstruct the specific physical and chemical contributions driving the multi-stage transformation of the Cuf, the total potential energy was deconvoluted into its constitutive energies components associated to the different chemical bonds and features (Fig. 4). This precise partition allows for a clear distinction between the non-bonding thermodynamic changes during chemical desorption and the bonding changes during chemical reduction.

**Fig. 4:**
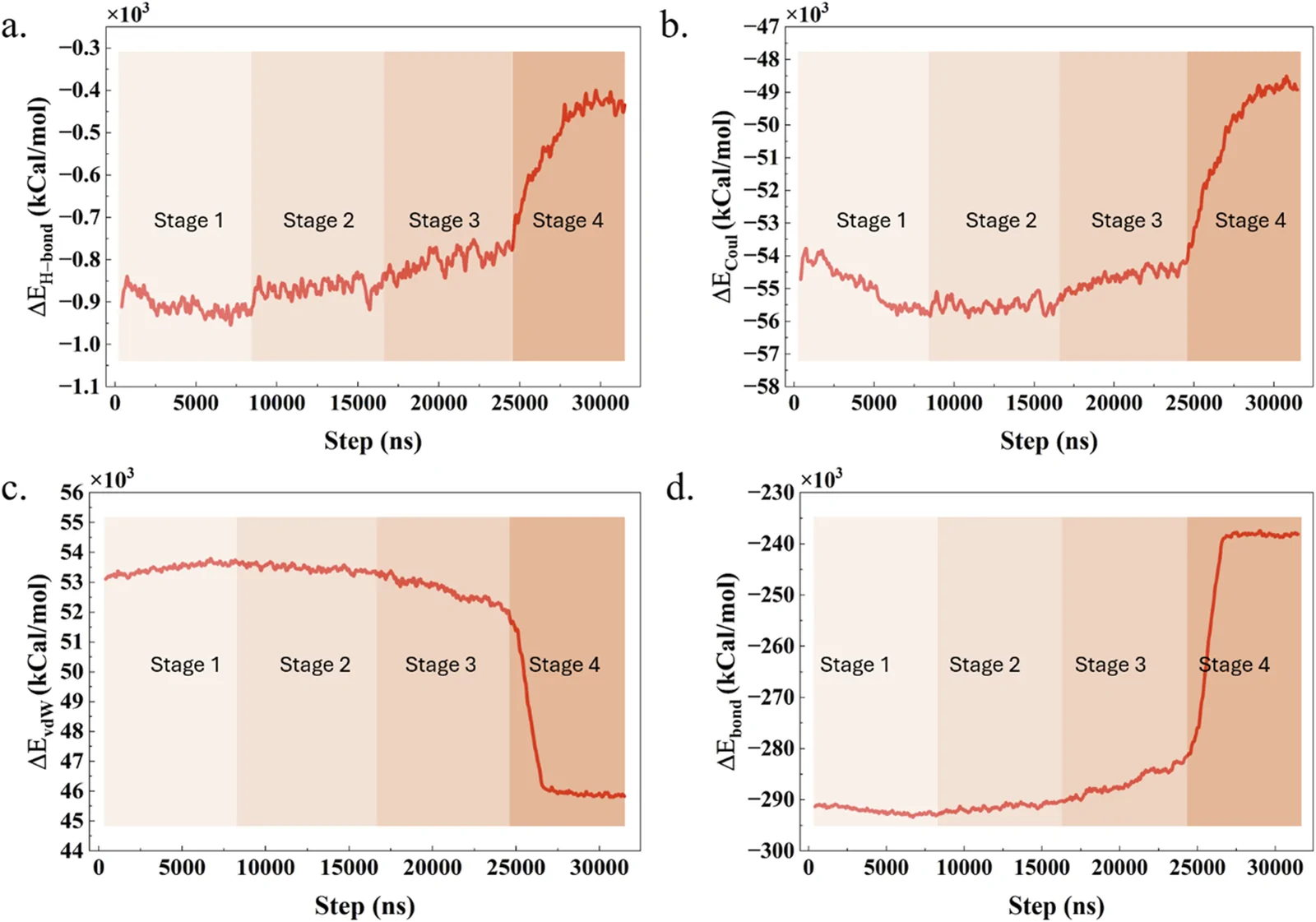
Evolution of specific intra- and intermolecular interaction energies during the MD simulation (from 298 K to 490 K). (a) Hydrogen-bond energy profile tracking the sequential disruption of the peripheral water network during the three desorption stages. (b) Coulombic and (c) van der Waals energy profiles capturing long-range electrostatic relaxation, changes in dielectric screening, and lattice contraction upon water loss. (d) Bond energy profile illustrating the stability of the core coordination sphere up to 375 K, followed by a sharp, discontinuity at 490 K that marks the cleavage of the final inner-sphere aquo-ligand and the subsequent decomposition.

During the sequential dehydration steps spanning from 298 K to 375 K (Table 1), the hydrogen-bond energy (Δ*E*_H-bond_) exhibits a step-like upward increase (Fig. 4a). This trend directly quantifies the energetic penalty of disrupting the cooperative hydrogen-bonding network that anchors the outer-envelope aquo-ligands within the crystalline structure. Concurrently, the coulombic energy (Δ*E*_coul_), which is related to the ionic bonds, and van der Waals (Δ*E*_vdW_) energies, which represents the mass forces, exhibit synchronous, localized fluctuations (Fig. 4b and c). These shifts are attributed to the modification of the long-range electrostatic and dielectric interactions within the unit cell as polar water molecules continuously escape into the vacuum. The structural relaxation matching the water desorption induces an isotropic lattice contraction, which manifests as a minor compaction signature in the non-bonded attractive van der Waals terms.^30^

Crucially, throughout these early desorption regimes, the bond energy (Δ*E*_bond_), which is associated to bond order, type, number, and length, remains almost constant, maintaining a nearly stable plateau^30^ (Fig. 4d). This energetic invariance provides unequivocal atomistic proof that the primary coordination sphere comprised of the equatorial formate chelation and the axial inner-sphere aquo-ligand is thermodynamically protected against peripheral lattice deformation. The initial loss of 75% of the total hydration water molecules is therefore confirmed to be a purely chemical desorption process that lacks the structural power to perturbate the core coordination geometry or electronic integrity of the central Cu^2+^ cations.

This localized stability is dramatically broken upon transitioning to the final thermal stage at 490 K. At this activation threshold, the bond energy (Δ*E*_bond_) undergoes a rapid asymptotic ascent that subsequently flattens into a new stable plateau (Fig. 4d). This energetic surge marks the destabilization and definitive breakage of the primary inner-sphere coordination bonds, specifically the high-energy Cu–O bonds binding the fourth aquo-ligand and the surrounding formate groups.^31,32^ Coinciding exactly with this covalent disruption, the coulombic energy increases sharply (becoming less negative) before also levelling off into a plateau, reflecting a complete redistribution of localized atomic charges. As the Electronegativity Equalization Method (EEM) captures the rapid charge transfer associated with the reduction of Cu^2+^ to Cu^0^ (see Fig. 5 in the next section), the long-range ionic interactions of the lattice are structurally extinguished.

**Fig. 5:**
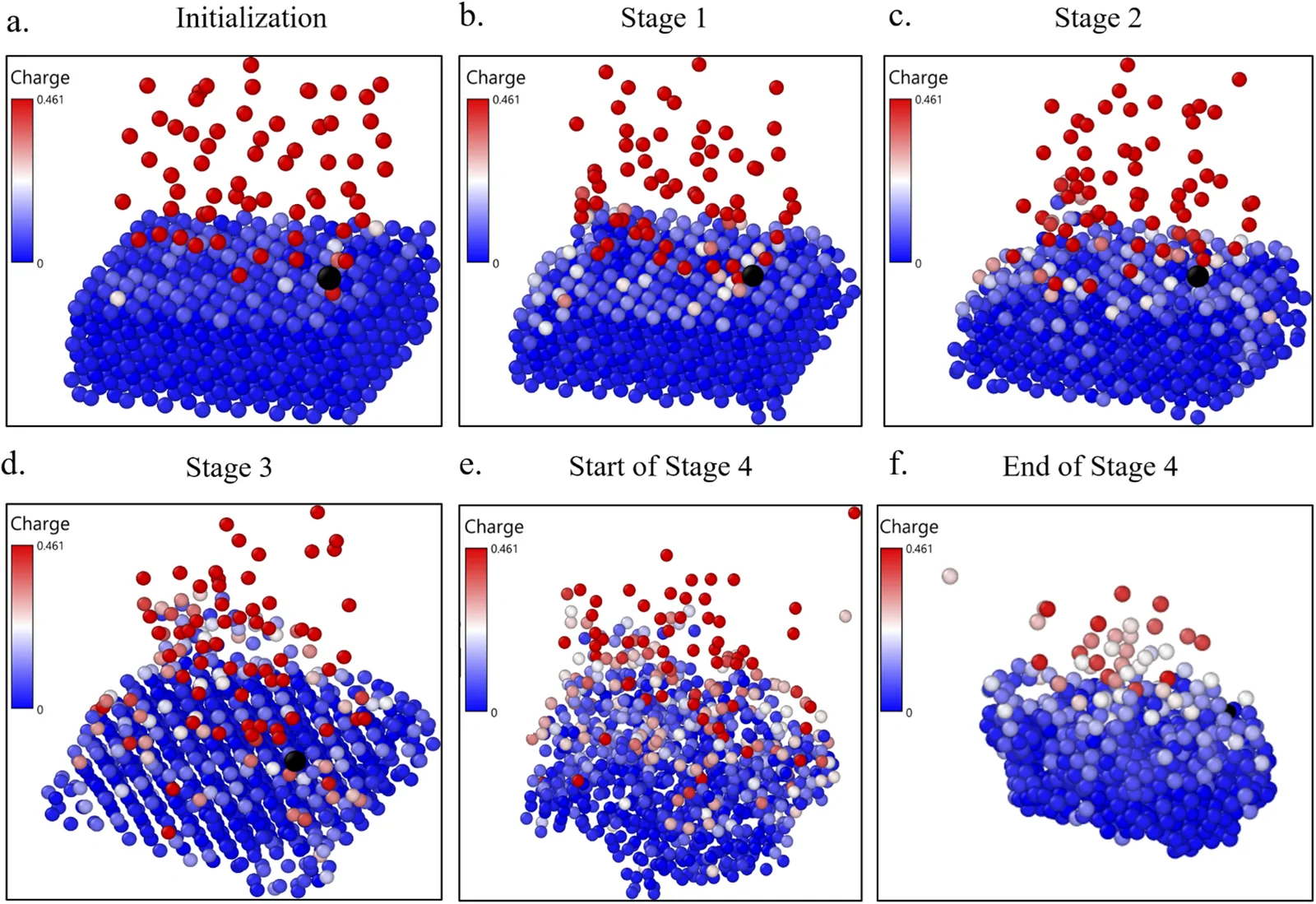
Dynamic charge equilibration tracking the electronic reduction of Cu^2+^ to Cu^0^ during the MD simulation. Color scales indicate atomic charge, where red spheres represent a positive charge (+0.461, ionic Cu^2+^) and blue spheres represent a neutral charge (0, metallic Cu^0^). All the colors in between represent transition states. (a) Initial state at 298 K, showing the highly charged ionic precursor lattice resting on the neutral Cu substrate. (b–d) During the progressive dehydration stages (325 K, 353 K, and 375 K), the precursor Cu atoms retain their original +0.461 oxidation state (remaining red), indicating that partial dehydration does not initiate reduction. (e) At the start of stage 4 (decomposition onset at 490 K), the structural collapse induced by the release of the final water molecule triggers a distinct electronic shift, transitioning precursor copper atoms toward a neutral charge state. (f) By the end of stage 4, the precursor fully reduces and agglomerates into a neutral Cu^0^ phase (predominantly blue), successfully simulating the *in situ* formation of metallic Cu. The black sphere is the referencing cursor.

The simultaneous collapse of the remaining hydrogen-bonding states (Fig. 4a) and the sharp drop in the non-bonded van der Waals profile (Fig. 4c); both of which similarly stabilize into plateaus, confirm the transformation from an ordered crystalline structure into an amorphous, phase-separated system consisting of metallic Cu clusters and released volatile gases (CO_2_ and H_2_). The emergence of these concurrent plateaus across all energetic components in stage 4 indicates that the chemical reduction has reached completion and the fully decomposed system has settled into a new thermodynamic equilibrium. Secondary structural correction terms including valence angle, atom, charge equilibration and lone-pair energies, similarly maintain stable baselines until this decomposition threshold is breached, as detailed in Fig. S3 in SI.

### Charge distribution and electronic transitions of Cu in Cuf

To describe the chemical mechanics driving the precursor reduction, the instantaneous partial atomic charge evolution of the Cu centers was continuously monitored *via* the EEM (Fig. 5). While the EEM/QEq method provides empirical partial atomic charges rather than formal integer oxidation states, this dynamic charge-tracking protocol provides real-time visibility into the relative electron distribution and transient redox trajectories as the coordination environment experiences thermal stress. At the baseline initialization temperature of 298 K, the Cu atoms within the pristine Cuf precursor lattice exhibit a highly localized, positive partial charge distribution averaging +0.461 (represented visually as red spheres in Fig. 5a). This well-defined electronic signature clearly differentiates the ionic, hexa-coordinated Cu^2+^ nodes within the framework from the zero-valence metallic Cu^0^ atoms constituting the underlying β-Cu substrate (represented as blue spheres, anchoring the lower electronic boundary). As the thermal simulation progresses through the sequential desorption regimes from 325 K to 375 K (Fig. 5b–d), the mean partial atomic charge of the framework Cu ions remains remarkably invariant, maintaining its highly electropositive baseline. This electronic rigidity strongly corroborates the thermodynamic trends detailed previously. The chemical desorption of the three outer-lattice hydration water molecules induces macro-structural dilatation and local lattice warping but lacks the perturbative capacity to alter the primary coordination geometry or destabilize the local electrostatic topology of the Cu^2+^ centers.

Conversely, upon transitioning to the final thermal activation stage at 490 K, the intermediate monohydrate framework undergoes an abrupt structural collapse that triggers a rapid, non-monotonic electronic redistribution at the onset of decomposition (Fig. 5e). This sharp alteration in partial atomic charge is temporally and spatially synchronized with the cleavage of the final inner-sphere Cu–O aquo-ligand bond and the concurrent decarboxylation of the formate groups. As the localized coordination sphere disassembles, a ligand-to-metal charge transfer (LMCT) is initiated. Visually, this transient redox pathway is captured by the color migration of the precursor Cu atoms, which transition rapidly from highly electropositive red, through a white/light blue intermediate transition state (Fig. 5e), and finally into a predominantly blue zero-valence metallic Cu^0^ phase at the end of stage 4 (Fig. 5f). This intermediate simulation state correlates with the transient Cu(i) species widely reported in macroscopic thermochemical studies of Cu(ii) formate.^33^ The structural collapse and agglomeration of these neutralized atoms visually confirm that the reduction process has reached thermodynamic equilibrium. This real-time electronic trajectory provides definitive atomistic validation that the desorption of the fourth, tightly bound water molecule acts as the determining thermodynamic and electronic switch that lowers the activation barrier for chemical reduction, directly driving the *in situ* metallization of the MOD ink framework.

## Experimental

### Materials

Cu(ii) formate tetrahydrate (Cuf), Cu(HCOO)_2_·4H_2_O, 98%, was purchased from Thermo Fisher Scientific, USA, and used as received.

### Thermal analysis and mass spectrum of Cuf

To set up the thermal boundary conditions for the MD simulations, the dehydration and decomposition profile of Cuf was measured experimentally. Heat flow and mass loss of Cuf were analyzed using differential scanning calorimetry (DSC 821 from Mettler Toledo, USA) and thermal gravimetric analysis (TGA/DSC 3+ from Mettler Toledo, USA). In addition to DSC and TGA measurements, mass spectroscopy of the Cuf was carried out using QMS 403 Aeolos Quadro analyzer system from Netzsch, Germany. The samples were measured under nitrogen atmosphere (N_2_ flow rate: 30 mL min^−1^) with a heating rate of 10 °C min^−1^ up to 250 °C followed by an isothermal holding at 250 °C for 5 min. The obtained values of the transition temperatures were also used to validate the simulation model.

### Simulation model construction

Once the key transition temperatures were identified, an atomistic simulation model was built. Initial atomic coordinates were generated based on a fully periodic three-dimensional monoclinic crystal lattice with 54 atomic sites per unit cell, using two-dimensional crystallographic data from previous studies, was created^26^ (see SI Fig. S4a and b). The 3D Cuf crystal structure was placed on a metallic β-Cu substrate, to provide a baseline for electronic state of metallic Cu. The specific fractional coordinates, atom designations, and initial charge parameters for the basis atoms are summarized in Table S1. Before applying heat, the system's energy was minimized and equilibrated (initialization) at room temperature (298 K) to set structural baseline and electronic parameters.^34^ Following this baseline equilibration at 298 K, the structural integrity of the simulated Cuf lattice was validated against experimental crystallographic data. The simulated Cu–O coordination bond lengths and monoclinic unit cell parameters remained in strong agreement with the baseline structures reported by Okada *et al.*,^26^ confirming the suitability of the selected ReaxFF potential for this specific metal–organic complex prior to thermal evaluation. Once initialization was completed, MD simulations were performed at four distinct temperature steps corresponding to the endothermic and exothermic peaks observed in the DSC profile.

### MD simulation parameters

The model evaluated the heating process to 325 K (52 °C), 353 K (80 °C) and 375 K (102 °C) to validate the stepwise release of the first, second and third hydration water molecule from the Cuf chemical structure, respectively. The final temperature step was performed at 490 K (217 °C) to evaluate the release of 4th water molecule and its role in the decomposition of the Cuf and reduction of Cu^2+^ to Cu^0^. The simulations were performed using the LAMMPS (Large-scale Atomic Molecular Massively Parallel Simulator) package. To accurately capture these chemical transformations, MD simulations were performed using the reactive force field (ReaxFF). ReaxFF utilizes a bond-order approach where breakage and formation of bonds are determined in an instantaneous localized environment which is suitable for simulating decarboxylation reaction and Cu(ii) reduction. The potential parameters for Cu/C/H/O interaction were taken from the optimized training sets developed by van Duin *et al.*, specifically calibrated for metal-oxide and metal–organic interfaces.^35–37^

The simulation domain was modelled in three dimensions (3D) using the full atom style and real units (with a timestep of 1.0 fs). Because the generated Cuf complex was placed on a metallic β-Cu substrate and modelled as a finite slab bounded by a large vacuum volume to accommodate gaseous desorption products, which dynamically escape of the interested region, fixed and non-periodic boundary configuration (f f f) was utilised in the simulation cell. This boundary configuration enables to remove the atoms that cross the sides of the cell (LAMMPS manual), realistically representing the escape of the gaseous products. The composite system consisted of a Cu formate tetrahydrate molecular structure (designated as the homogenous group) and a Cu substrate (modelled as a multi-layered workpiece containing Newtonian, thermostatic, and fixed boundary atom groups) (see SI Fig. S4c and d). The total simulation domain contained 2538 atoms, with the Cu formate tetrahydrate molecular layer comprising 1674 atoms and the substrate comprising 864 atoms. The initial physical dimensions of the substrate region were bounded from −2 to 95.5 Å in *x*, −8 to 89.5 Å in *y*, and −62 to −1 Å in *z*, while the Cu formate domain extended from *z* = 1 to 62.3 Å. The total simulation box was subsequently expanded to a large vacuum volume spanning from −100 to 200 Å in *x* and from −200 to 200 Å in both *y* and *z* directions to accommodate gaseous desorption products. Temperature control was maintained dynamically across the active regions by coupling an *NVE* time-integration scheme (constant number of particles, *N*, constant volume, *V*, and constant energy, *E*) with a Langevin thermostat (damping parameter of 1.0 fs).

During these temperature steps, multiple atomic-level interaction energies including covalent bonding, lone-pair interactions, and coulombic energies were monitored *via* the pair reax/c compute to evaluate crystal stability and bond dissociation pathways. Because MD operates on a femtosecond-to-nanosecond timescale compared to the minutes-long macroscopic profiles of experimental heating, higher heating rates were necessarily employed. This temporal discrepancy can cause a slight upward shift in transition temperatures relative to experimental data. To mitigate these timeline effects and accurately model charge transfers, the Electronegativity Equalization Method (EEM) was integrated within ReaxFF *via* a charge equilibration fix (qeq/reax). While standard EEM/QEq effectively captures the localized charge migration during ligand dissociation at the dense substrate interface, it is noted that conventional QEq can overestimate long-range charge transfer in rapidly fragmenting gaseous systems (*e.g.*, releasing CO_2_ and H_2_) in vacuum.^38^ Because the primary reduction tracking focuses on the interfacial agglomeration of Cu, these long-range artefacts regarding the volatile by-products are minimized, though they remain an inherent limitation of the standard formulation. This approach facilitated the direct observation of electronic shifts associated with the Cu transition (Cu^2+^ to Cu^0^) during ligand reorganization and structural collapse. Post-simulation visualization of the atomistic trajectories and dynamic charge distributions was performed using the Open Visualization Tool (OVITO).^39^ All thermodynamic and energy profile graphs were generated and analyzed using Origin software (OriginLab Corporation).

## Conclusions

By integrating macroscopic thermal analyses with MD simulations, this study provides a comprehensive mechanistic understanding of Cu(ii) formate tetrahydrate (Cuf) decomposition. The findings clearly distinguish between physical water desorption and chemical structural decomposition processes which are governed by the binding energies of four hydration water molecules within the Cuf crystal lattice.

The initial three-step dehydration process systematically removes the outer, hydrogen-bonded water lattice. The simulations confirm the chemical desorption process where the Cu^2+^ oxidation state remains unchanged (positive charge of +0.461) because the inner coordination sphere comprising of formate ligand and the final water molecule remains structurally intact. The critical transition is governed by the fourth water molecule of hydration, which is tightly coordinated to the Cu^2+^ ion. Its release at 490 K (217 °C) represents the determining step for the loss of structure. The disruption of the organised hydration shell into bulk water changes the coordination geometry from a stable complex to an unstable state, sterically and electronically destabilizing the surrounding formate ligands.

The dynamic charge equilibration tracking demonstrates that the reduction of Cu^2+^ to Cu^0^ is not a gradual process but an electronic shift triggered by the disassembly of the highly ordered network of the water molecules. Once the Cu–O bond (with the 4th water molecule of hydration) breaks, the formate ligand undergoes rapid decarboxylation, releasing CO_2_ and H_2_. Furthermore, the thermodynamic energy plateaus and visual charge tracking at the end of the simulation confirm the reaction's completion, as the fully reduced, Cu^0^ atoms successfully agglomerate into a stable, phase-separated metallic cluster. The model suggests that the 4th water molecule does not merely leave, but its release actively facilitates the electron transfer required for Cu^2+^ reduction. The model suggests that the fourth water molecule does not merely leave, but its release acts as a stoichiometric structural trigger that facilitates the electron transfer required for Cu^2+^ reduction. This observation provides an atomistic explanation for the sharp exothermic peaks detected by DSC experiments.

These insights establish a framework for the rational design of low-temperature Cu MOD inks for applications in flexible printed electronics. A key factor of the study was the demonstration that the reduction to Cu^0^ is contingent upon the destabilization of the inner hydration shell. To lower the processing temperature, which is crucial for heat-sensitive substrates like PET, the ink design must consider complexing ligands (*e.g.*, alkanolamines) specifically for their ability to:

- Displace the 4th water molecule more efficiently than the native formate structure;

- Lower the binding energy of the core Cu-complex through steric hindrance or ligand denticity.

By quantifying these interactions through the MD framework established here, it becomes possible to predict the decomposition behavior of complex Cuf–amine systems, enabling the development of high-conductivity, low-cost inks optimized for sustainable electronics manufacturing.

Finally, although this investigation provides significant insight and valuable information for understanding of the copper formate sintering mechanism, the effect of higher temperatures on the chemical and physical phenomena involved in this process was absent in the present study. These simulations conducted at higher temperature will therefore be undertaken in future studies, giving their potential applications and the prospect of providing further significant scientific insight into copper formate sintering mechanism.

## Author contributions

N. M. and F. C. conceptualized the study. N. M., J. I. A.-T., and G. E. conducted the investigation and validated the results, while N. M., F. C. and G. E. curated the data. All authors contributed to the formal analysis and methodology. N. M. and J. I. A.-T. developed the software, prepared the visualizations, and wrote the original manuscript draft. H. K. and G. E. acquired funding. H. K., F. C., and G. E. provided resources and reviewed and edited the manuscript. Supervision was provided by J. I. A.-T., H. K., F. C., and G. E., with G. E. also handling project administration. All authors reviewed the manuscript.

## Conflicts of interest

There are no conflicts to declare.

## Supplementary Material

RA-OLF-D6RA07869A-s001

## Data Availability

The LAMMPS input scripts, the ReaxFF potential files used for the molecular dynamics simulations, and the data that support the findings of this study are available from the corresponding authors on request. The data supporting this article have been included as part of the supplementary information (SI). Supplementary information is available. See DOI: https://doi.org/10.1039/d6ra07869a.
