# Supplementary material for: Decomposition process of copper(ii) formate tetrahydrate through molecular dynamics simulations
Source: RSC Adv. 2026 Oct 16;16(47):63327–37. doi: 10.1039/d6ra07869a (PMC13673690; doi:10.1039/d6ra07869a)
Supplement: Supplementary file 1 [file RA-016-D6RA07869A-s001.pdf]

Supplementary Information (SI) for RSC Advances.
This journal is © The Royal Society of Chemistry

Decomposition process of copper(II) formate tetrahydrate through molecular dynamics simulations

Nihesh Mohan, *[a] Juan Ignacio Ahuir-Torres, [b] Hiren Kotadia, [b] Fosca Conti *[c] and Gordon Elger[a]

[a] *Institute of Innovative Mobility (IIMo), Technische Hochschule Ingolstadt, Esplanade 10, 85049, Ingolstadt, Germany.*

[b] *School of Engineering and Built environment, Liverpool John Moores University, Liverpool, L3 3AF, UK.*

[c] *Department of Chemical Sciences, University of Padova, Padova, 35131, Italy.*

* Corresponding authors: fosca.conti@unipd.it and nihesh.mohan@thi.de

Supplementary data

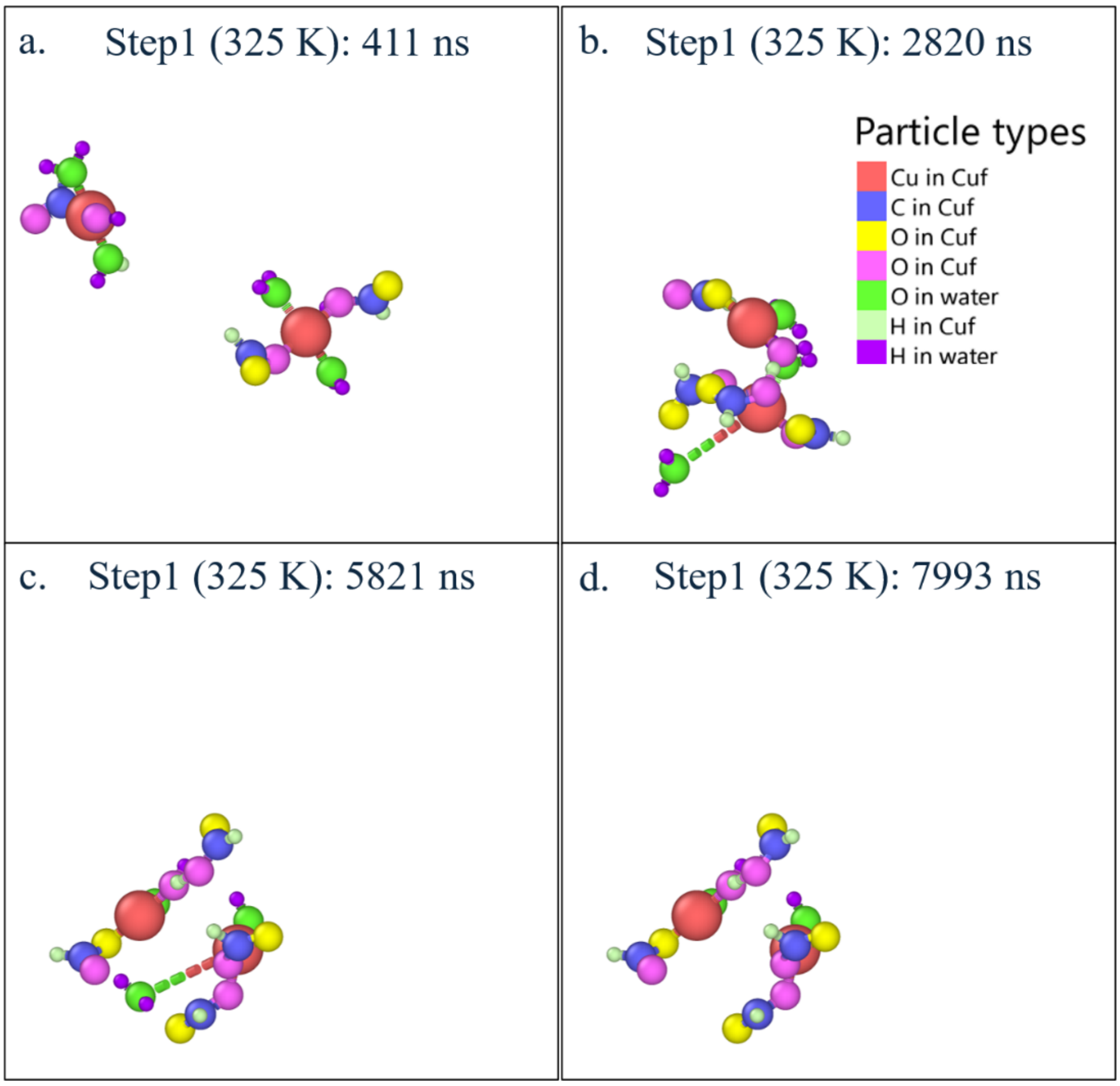

Fig. S1 Atomistic tracking of hydration water detachment from the copper formate coordination shell at 325 K (52°C). **(a) 411 ns**: Initial state showing two distinct copper centers with their complete, intact hydration shells. **(b) 2820 ns**: Under the influence of localized thermal fluctuations, a specific copper-water coordinate bond begins to stretch, initiating the outward displacement of the target water molecule. **(c) 5821 ns**: Transition state highlighting the final breaking of the coordinate bond (indicated by the dashed green line) as this specific water molecule physically detaches from the copper center. **(d) 7993 ns**: Complete detachment and spatial escape of the freed water molecule, leaving a partially dehydrated copper center behind while neighbouring water molecules remain bound.

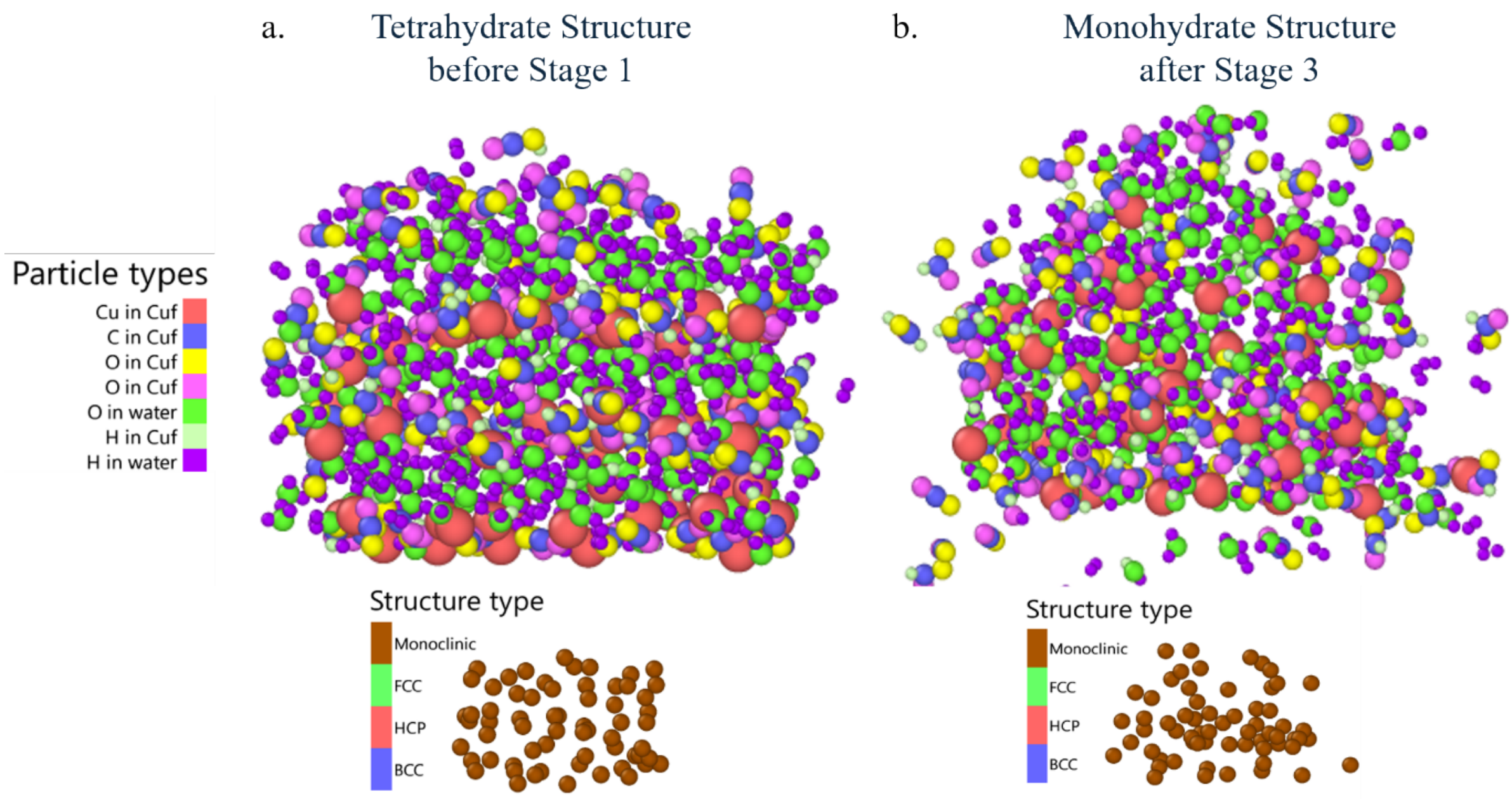

Fig. S2 Atomistic structural comparison and lattice stability verification of copper formate ($Cu(HCOO)_2$) during stepwise dehydration.

(a) Tetrahydrate structure before Stage 1:

Top: Molecular dynamics (MD) configuration of the initial, fully coordinated copper formate tetrahydrate (Cu(HCOO)₂·4H₂O) supercell, showing the highly dense and interconnected hydrogen-bonded network of hydration water (Oxygen: green; Hydrogen: purple) within the precursor framework. **Bottom**: Quantitative Polyhedral Template Matching (PTM) of the isolated copper sublattice, classifying 100% of the copper atoms as Monoclinic (brown spheres), confirming a highly ordered, non-metallic crystalline coordination baseline.

(b) Monohydrate structure after Stage 3:

Top: The resulting Cu(HCOO)₂ · 1H₂O configuration, highlighting the extensive physical removal of volatile hydration water molecules while the primary metal-organic skeleton remains spatially grouped. **Bottom**: Corresponding PTM analysis showing that despite removal of ¾ of water of hydration from the crystal lattice, 100% of the copper atoms remain as Monoclinic (brown spheres) with 0% transition to metallic close-packed phases (FCC, HCP, or BCC). This directly validates that the bulk lattice maintains its structural coherence and resists premature thermal collapse or reduction.

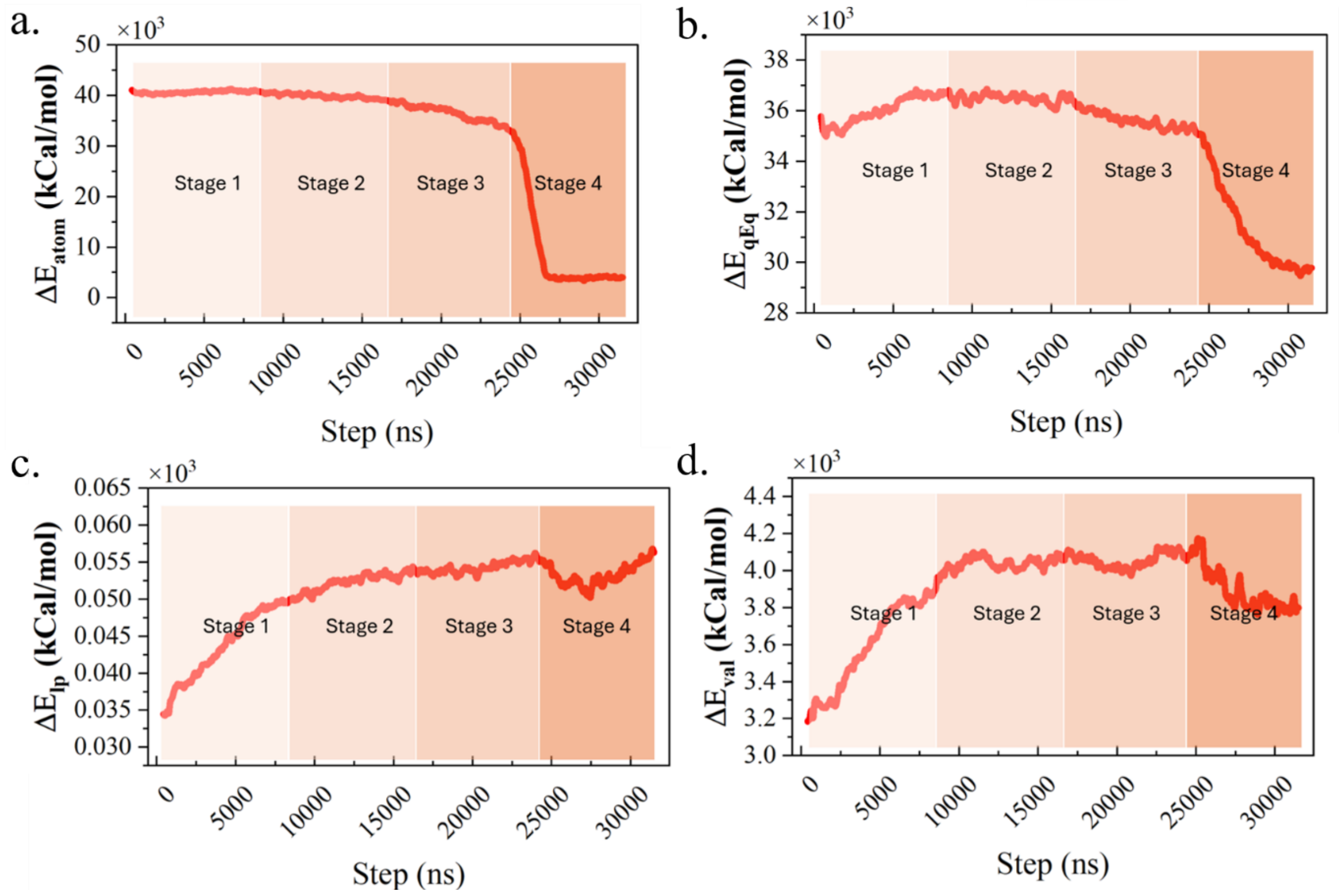

Fig. S3 Evolution of secondary geometric, electronic, and charge equilibration energy components within the ReaxFF framework from 298 K to 490 K. **(a)** Atom energy (ΔE_{atom}) and **(b)** charge equilibration energy (ΔE_{qEq}) profiling the ultimate thermodynamic stabilization and electrostatic relaxation of the system during in-situ metallization at 490 K. **(c)** Lone-pair energy (ΔE_{lp}) tracking orbital symmetry shifts. **(d)** Valence angle energy (ΔE_{val}) showcasing local conformation strain accumulation during desolvation.

Description:

To provide a comprehensive accounting of the thermodynamic trajectory computed via the ReaxFF reactive force field, the evolution of secondary geometric, electronic, and electrostatic relaxation energy components was rigorously monitored (**Fig. S3**). These secondary parameters capture local conformational strains and polarization costs that directly complement the primary interaction criteria detailed in the main manuscript. During the initial thermal desolvation windows spanning 298 K to 375 K, the valence angle energy (ΔE_{val}) rises steeply before establishing a high-energy plateau, quantifying the localized bending strain imposed on the O-Cu-O and O-C-O coordination geometries as the unit cell experiences anisotropic lattice dilatation to accommodate the evacuation of the first three aquo-ligands. Upon reaching the critical 490 K (217°C) thermochemical threshold, both profiles exhibit profound, non-linear discontinuities; the sudden drop and severe fluctuations signify the catastrophic mechanical collapse of rigid structural angles and the simultaneous relief of torsional constraints via multi-body molecular framework dissociation. Concurrently, local electronic and orbital reconfigurations are explicitly indexed by the lone-pair energy correction (ΔE_{lp}), which exhibits a steady, monotonic increase across the desolvation regime. Within the ReaxFF formalism, this term accounts for electronic penalties associated with under-coordinated or distorted valence architectures, directly tracking the shifting electronic demand and localized loss of orbital symmetry around the oxygen nodes as coordinate Cu-O_{water} bonds are systematically severed. This progressive accumulation of local instability culminates at the 490 K threshold, where the system undergoes an abrupt thermodynamic restructuring marked by sharp

shifts in both the atom baseline energy (ΔE_{atom}) and the charge equilibration energy (ΔE_{qEq}). The precipitous drop in ΔE_{atom} represents the favourable thermodynamic transition of highly strained, bonded complex precursors into a phase-separated state comprising cohesive metallic Cu° clusters and liberated volatile gas molecules (CO_2 and H_2). Simultaneously, the sharp minimization of ΔE_{qEq} indicates that the system resolves its long-range electrostatic polarization penalties via a definitive ligand-to-metal charge transfer (LMCT), proving that the catastrophic framework breakdown effectively drives the electronic stabilization and in-situ metallization of the system.

Supplementary Table S1: Fractional coordinates, atom types, and initial partial charges for the 54-atom basis of the monoclinic copper(II) formate tetrahydrate (Cuf) crystal lattice.

Basis Index (M)	Fractional Coordinates (x, y, z)			Basis atom designation	Atom Type (charge) – iType	Basis values
Basis 1	0	0	0	Cu	Cu (0.461) – 1	Basis 1 1
Basis 2	0.237	0.269	0.024	C	C (0.26) – 2	Basis 2 2
Basis 3	0.205	0.092	0.080	O_F (1)	O (-0.324) – 3	Basis 3 3
Basis 4	0.116	0.213	0.088	O_F (2)	O (-0.373) – 4	Basis 4 4
Basis 5	0.430	0.401	0.641	O_w(1)	O (-0.55) – 5	Basis 5 5
Basis 6	0.089	0.357	0.482	O_w(2)	O (-0.55) – 5	Basis 6 5
Basis 7	0.207	0.692	0.071	H_F	H (0.1) – 6	Basis 7 6
Basis 8	0.311	0.385	0.592	H (1)	H (0.35) – 7	Basis 8 7
Basis 9	0.481	0.296	0.592	H (2)	H (0.35) – 7	Basis 9 7
Basis 10	0.473	0.462	0.518	H (3)	H (0.35) – 7	Basis 10 7
Basis 11	0.084	0.320	0.339	H (4)	H (0.35) – 7	Basis 11 7
Basis 12	0.029	0.475	0.480	H (5)	H (0.35) – 7	Basis 12 7
Basis 13	0.195	0.431	0.585	H (6)	H (0.35) – 7	Basis 13 7
Basis 14	0.040	0.269	0.533	H (7)	H (0.35) – 7	Basis 14 7

Description:

This table provides the initial structural and electronic parameters used to construct the 3D Cuf$ supercell for ReaxFF molecular dynamics simulations. The fractional coordinates (x, y, z) define the positions of the atoms within the monoclinic unit cell. **O_F** designates oxygen atoms belonging to the formate ligands, while **O_w** designates oxygen atoms within the hydration water molecules. Similarly, **H_F** refers to the hydrogen atom in the formate group, and **H(1–7)** refers to the various hydrogen positions within the water network. The **iType**

column represents the specific atom type assigned within the ReaxFF framework to distinguish between different chemical environments, and the initial charges were calculated using the Electronegativity Equalization Method (EEM) to establish the Cu^{2+} baseline.

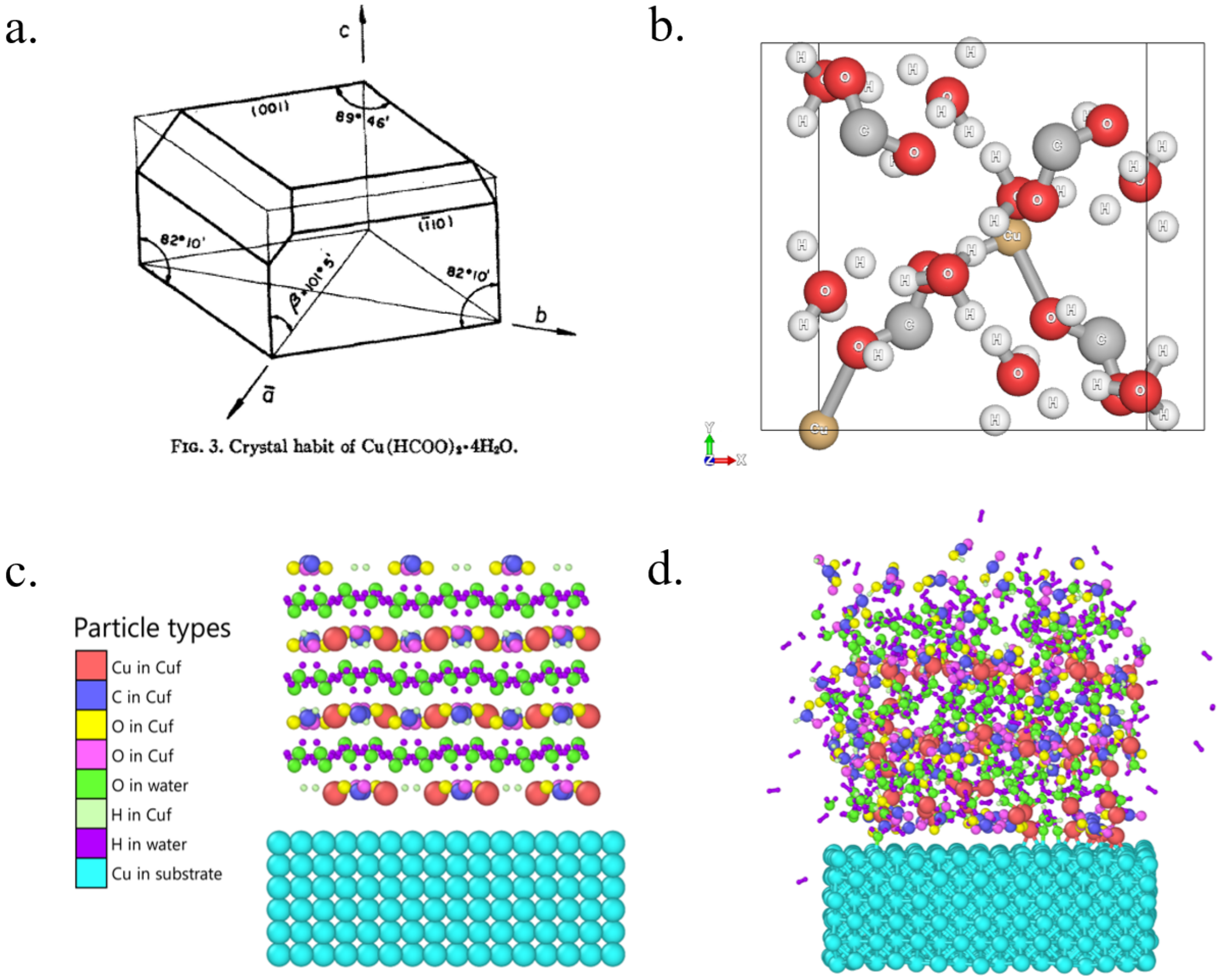

Fig. S4 Construction and initialization of the atomistic simulation model. **(a, b)** Conversion of the Cu(II) formate tetrahydrate crystal lattice into three-dimensional monoclinic space lattice coordinates (54 atomic sites per unit cell). The 2D crystallographic data was sourced from Okada et al. [1], and the space lattice was derived using DensityFlow software (https://www.densityflow.com/en/unit2prim.php). (c) The constructed composite material model comprising the Cu(II) formate tetrahydrate (Cuf) crystal and the metallic Cu substrate prior to initialization. (d) The atomistic configuration of the system at the start of the MD simulation, following the completion of the energy minimization and initialization phase. Atom colors correspond to the particle types defined in the embedded legend.

[1]. K. Okada, M.I. Kay, D.T. Cromer, I. Almodovar, Crystal structure by neutron diffraction and the antiferroelectric phase transition in Copper Formate Tetrahydrate, J. Chem. Phys. 44(4) (1966) 1648–1653. https://doi.org/10.1063/1.1726904
